# Accuracy and outcome of mandibular fracture reduction without and with an aid of a repositioning forceps

**DOI:** 10.1007/s10006-019-00759-0

**Published:** 2019-05-04

**Authors:** Enkh-Orchlon Batbayar, Somaia Malwand, Pieter U. Dijkstra, Ruud R. M. Bos, Baucke van Minnen

**Affiliations:** 1 Department of Oral and Maxillofacial Surgery, University Medical Center Groningen, University of Groningen, P.O. Box 30.001, 9700RB Groningen, The Netherlands; 2 Department of Rehabilitation, University Medical Center Groningen, University of Groningen, P.O. Box 30.001, 9700RB Groningen, The Netherlands

**Keywords:** Mandibular fracture, Open fracture reduction, Surgical instruments, Postoperative complications

## Abstract

**Purpose:**

It is presumed that adequate reduction of a fracture of the mandible favors bone healing and diminishes the risk of complications. In this retrospective study, we compared the accuracy of fracture alignment and complication rate of mandibular fractures reduced without or with aid of a repositioning forceps.

**Methods:**

Retrospective analysis of consecutive 252 patients with mandibular fractures treated between January 2010 and December 2016. Eligible for this study were patients with isolated mandibular fractures needing open reduction and internal fixation in whom pre- and postoperative radiographs and patient records were available. In total, 131 (252 fractures) patients fulfilled the inclusion criteria.

**Results:**

Seventy-one (54%) patients were men. Mean age of the patients was 33 ± 16.5 years, and the median and interquartile range of age was 25 (20;41). In 54 patients, mandibular fractures were reduced without the aid of repositioning forceps, and in the remaining 77 patients, the fractures were reduced with the aid of the repositioning forceps. Anatomical alignment of the fractures was poor in the non-forceps-aided group (48%) compared to the forceps-aided group (58%) (*P* = .067). Overall complication rate was higher in the group of fractures reduced without the aid of forceps (17%) than in the forceps-aided group (7%) (*P* = .045; OR, 2.7; 95% CI, 1.0–7.4).

**Conclusions:**

Mandibular fractures reduced with the aid of repositioning forceps are accompanied by a lower complication rate and better alignment. This is an important observation as better alignment of the fracture fragments favors bone healing and reduces complications.

## Introduction

Mandibular fracture treatment aims to achieve adequate reduction of the fracture fragments, to immobilize these fragments firmly in order to restore premorbid occlusion and to promote direct bone healing. Common methods for reduction of mandibular fractures include intermaxillary fixation (IMF), manual reduction, and the use of a repositioning forceps. After adequate reduction, the aligned fragments are fixated with osteosynthesis materials.

IMF is used primarily to restore occlusion and secondarily to reduce the fracture [1, 2]. Commonly, IMF is applied by wiring the upper and lower jaws with the arch bars, but there is a variety of alternative techniques including IMF screws. Although shown to be successful, the various IMF techniques have drawbacks including an increased risk of root injury, IMF screw failure, accidental needle stick injury, and discomfort to the patient [3–5]. The use of IMF is not a prerequisite to reduce and fixate mandibular fractures [6, 7], and manual reduction and use of repositioning forceps are reliable alternatives [2, 8–13]. When performing manual reduction, extra hands to reduce the fracture fragments are needed, preferably with aid of a skilled assistant [3]. Moreover, there is not always sufficient room to insert osteosynthesis materials via intraoral approach due to the limited access to the fracture when manually aligning fracture fragments. With a repositioning forceps, a more accurate anatomical reduction and higher pre-compression can be achieved compared to IMF or manual reduction [2]. This better alignment of the fragments is presumed to favor bone healing and diminish risks of complications.

All abovementioned mandibular fracture reduction techniques are viable options for treatment of mandibular fractures. In clinical practice, these techniques are often used in combination with each other. The objective of the current study was to analyze the added value of using repositioning forceps in the mandibular fracture treatment. We hypothesize that mandibular fracture treatment, when a repositioning forceps is used, will result in a more accurate fracture alignment and less postoperative complications.

## Materials and methods

### Study population

The medical records of all patients who received surgical treatment for mandibular fractures between January 2010 and December 2016 at the Department of Oral and Maxillofacial Surgery, University Medical Centre Groningen, the Netherlands, were assessed for eligibility. Inclusion criteria were isolated mandibular fractures that required treatment with open reduction and internal fixation (ORIF) and availability of pre- and postoperative radiographs and records. Both unilateral single/double fracture(s) and bilateral fractures of the mandible were included. Exclusion criteria were closed treatment of a mandibular fracture, bi-maxillary fractures, inadequate radiographs, and fractures older than 3 weeks at the time of treatment.

### Fracture reduction techniques and surgery

**Fig. 1:**
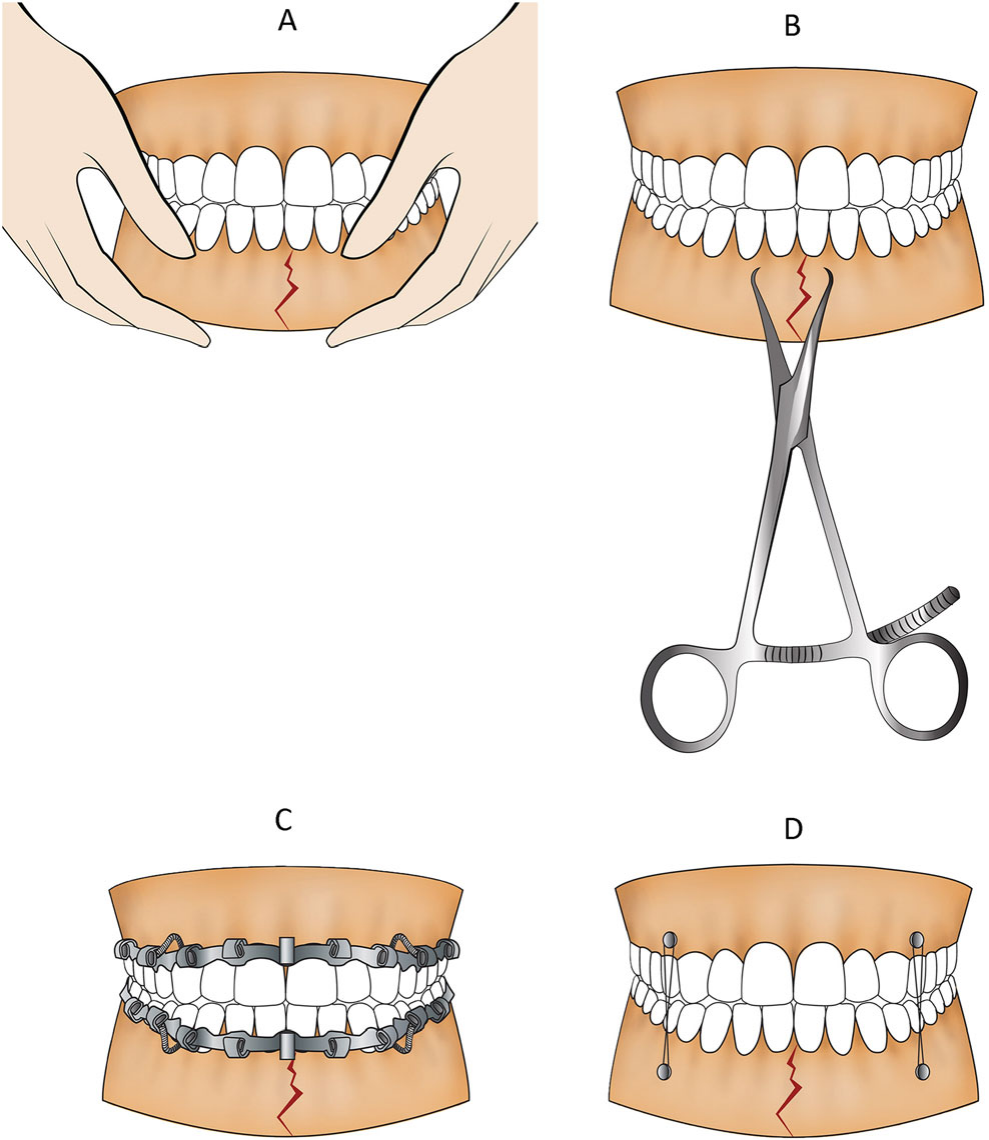
Illustrative explanation of the fracture reduction techniques. **a** Manual reduction. **b** Repositioning forceps. **c** IMF with arch bar and wire. **d** IMF screw and wire. The repositioning forceps-aided group includes **b** alone, or **b** combination with (and/or) **a**, **c**, and **d**. The non-forceps-aided group includes **a**, **c**, and **d** alone, or combination of those

Reduction of mandibular fractures was achieved by either manual reduction, IMF, the use of repositioning forceps, or a combination of these techniques (Fig. 1). The reduction techniques were grouped as follows: (1) fractures reduced without the aid of repositioning forceps, and (2) fracture reduced with the aid of repositioning forceps. All operations were performed under general anesthesia by an oral and maxillofacial surgeon assisted by residents. The choice of the reduction methods applied was based on the case complexity and the surgeon’s preference.

### Study variables and outcome measurements

Age, gender, cause of trauma, comorbidity, occlusal state, oral hygiene, smoking habits, dental status, fracture type, fracture location and type, the order in which IMF, manual reduction and/or the use of a reduction forceps were used, fracture fixation method, postoperative fracture alignment and complications were extracted from the patient records. Postoperative fracture alignment was evaluated by two observers based on postoperative radiographs along with the clinical appraisal of occlusion according to the following score: (1) poor reduction of the fracture needing reoperation; (2) fracture was reduced with slight dislocation, but clinically with a satisfying occlusion; and (3) reduction of the fracture with anatomic alignment. The reported complications were divided into complications needing reoperation (major) and complications not needing surgical intervention (minor). The immediate postoperative occlusion was assessed by oral and maxillofacial surgeons of the department by clinical examnation and assessing the patients’s experience of occlusal disturbance.

### Statistical analysis

We performed two groups of statistical analyses, one with the patient as unit of analysis, and one with the fracture as unit of analysis. Age, gender, cause of trauma, comorbidity, occlusal state, oral hygiene, smoking habits, dental status, fracture type, and complications were analyzed per patient. Fracture location and type, fracture fixation method, postoperative fracture alignment, and complications were analyzed per fracture. When unit of analysis was fracture, possible concomitant condylar or ramus fractures were excluded from the comparison of reduction methods regarding complications, if the concomitant fractures were treated closed. In another words, the comparison was made only with fractures that underwent ORIF.

Chi-square (exact test when actual or expected cell filling was not too low), *t* test, Mann–Whitney *U*, and logistic regression analysis were applied to analyze differences between reduction techniques regarding reduction accuracy (alignment), patient and fracture characteristic, and postoperative complications.

To identify possible confounding factors, we explored associations between complications (no, yes) and the following variables: age, gender (male, female), comorbidity (no, yes), smoking (no, yes), oral hygiene (good, poor), velocity of trauma (high, low), mandible fracture(s) concomitant with condyle (no, yes), fracture reduction techniques (with the aid of forceps, without the aid of forceps), fracture location, and fracture fixation methods (load sharing, load bearing, or combination). Statistical significance was set at *p* ≤ .05 and consistently evaluated using two-sided tests. Data were analyzed with IBM SPSS version 23.0.03 software.

## Results

### General characteristics of patients

**Fig. 2:**
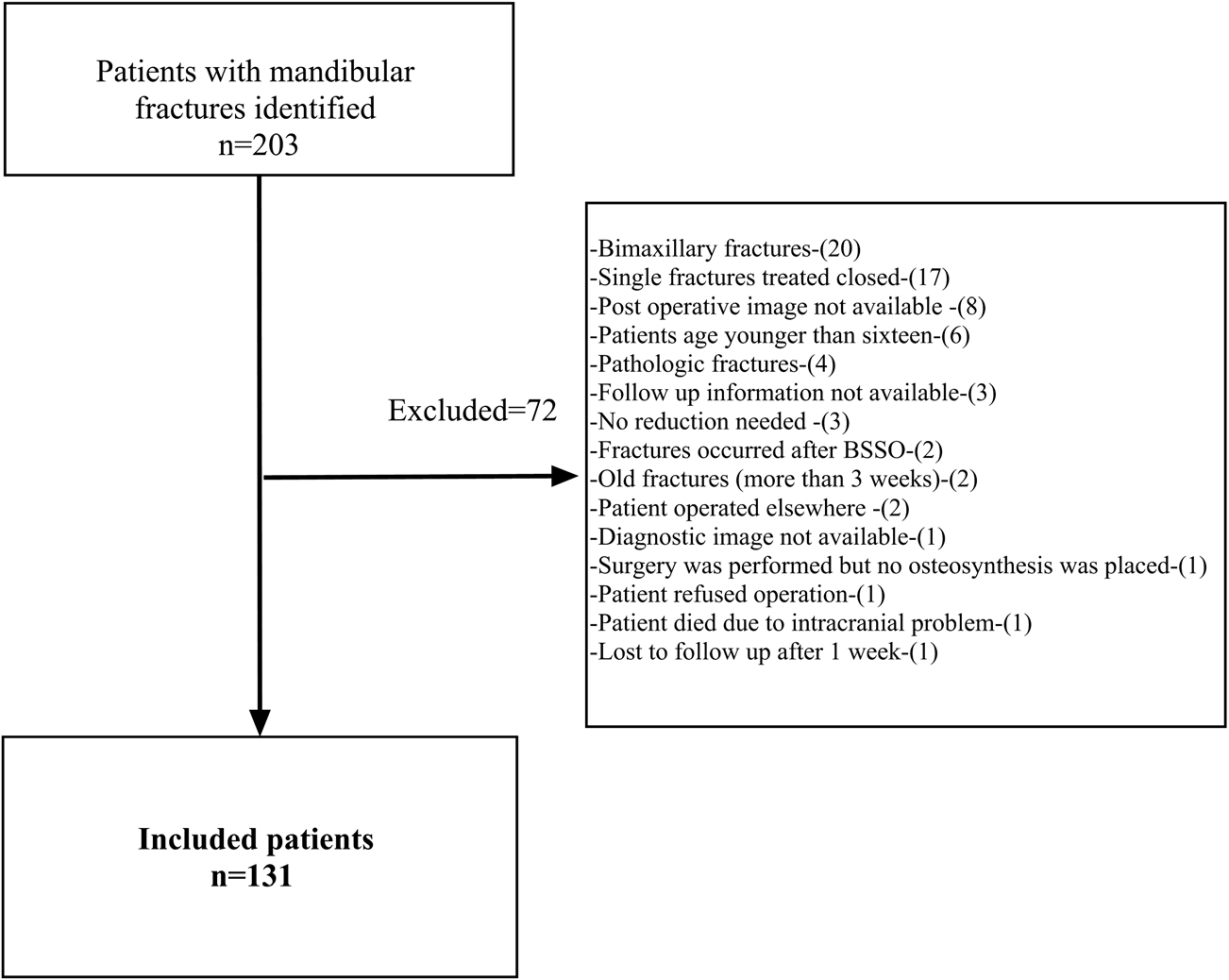
Flowchart of the patient selection process

Of the 203 patients with mandibular fractures that were treated between January 2010 and December 2016, 131 patients with 252 fractures met the inclusion criteria (Fig. 2). The mean age of the patients was 33 ± 16.5, the median (interquartile range (IQR)) of age was 25 (20;41), and 103 patients (79%) were male. The median (IQR) number of fractures per patients was 2 (1; 2). Three patients had cardiovascular disease, three patients had diabetes (type II), two patients had alcohol abuse, and one patient had a psychological problem.

Eighty-two (63%) out of the 131 eligible patients were fully dentate, 38 (29%) were partially dentate, and 11 (8%) patients were edentulous. Eighteen (14%) patients were smokers. Oral hygiene status was not recorded in 11 (8%) edentulous patients. In 110 (92%) patients, oral hygiene was good, and 10 (8%) patients had poor oral hygiene. The cause of the mandibular fractures was assault (*n* = 45, 34%), fall (*n* = 27, 21%), motor vehicle accident (*n* = 25, 19%), bicycle accident (*n* = 25 (19%), sports injury (*n* = 7, 5%), and accident at home (*n* = 2, 2%).

The fractures occurred in the mandibular (para)symphyseal region (*n* = 67, 27%), body (*n* = 54, 21%), angle (*n* = 47, 19%), ramus (*n* = 23, 9%), and condyle (*n* = 61, 24%). Among the 252 fractures, 206 (82%) fractures were simple, 37 (14%) comminuted and 9 (4%) incomplete. The simple fractures were more frequently observed in the (para)symphyseal (*n* = 53, 26%), angle (*n* = 42, 21%), and condyle or ramus (*n* = 75, 36%) region of the mandible, and less in the body region (*n* = 36, 17%). The comminuted fractures were mainly observed in the (para)symphyseal (*n* = 14, 38%) and body (*n* = 18, 48%) region followed by angle (*n* = 4, 11%) and condyle (*n* = 1, 3%) region.

### Patient characteristics and reduction techniques

In 54 patients, the fractures were reduced without the aid of a repositioning forceps, and in 77 (66%) patients, the fractures were reduced with the aid of the forceps. The two groups were similar with regard to age of the patients and number of mandibular fractures per patient (Table 1). Comorbidity was observed more often in the non-forceps-aided group (*P* = .04; OR = 3.6, 95% CI, 1–12).

**Table 1:** Patients’ characteristics and reduction techniques (unit of analysis: patients, *n* = 131)

		Reduction not aided by the forceps (*n* = 54)^a^	Reduction aided by the forceps (*n* = 77)^a^	*P* value^c^
Age (mean ± SD)		33.0 ± 16.4	32.9 ± 16.7	.687*
Number of fractures (mean ± SD)		1.9 ± 0.7	1.8 ± 0.7	.186*
Gender (*n* = 131)	Male	43 (80)	60 (78)	.496
	Female	11 (20)	17 (22)	
Smoking (*n* = 131)	No	49 (91)	64 (83)	.161
	Yes	5 (9)	13 (17)	
Oral hygiene (*n* = 120)	Good	44 (92)	66 (92)	.638
	Poor	4 (8)	6 (8)	
Comorbidity (*n* = 131)	No	45 (83)	73 (95)	.039
	Yes	9 (17)	4 (5)	
Dental status (*n* = 131)	Complete	37 (69)	45 (58)	.156
	Partially	11 (20)	27 (35)	
	Edentulous	6 (11)	5 (7)	
Fracture type (*n* = 131)	Single fracture	19 (35)	17 (22)	.432
	Single fracture concomitant condyle (unilateral or bilateral)	14 (26)	24 (31)	
	Bilateral or unilateral double fracture	19 (35)	32 (42)	
	Bilateral fracture concomitant condyle (unilateral or bilateral))	2 (4)	4 (5)	
Complication	No	39 (72)	71 (92)	.005
	Minor	10 (19)	3 (4)	
	Major	5 (9)	3 (4)	

^a^Column percentage

^b^Number of valid data (fractures treated with closed treatment excluded, no reduction was needed)

^c^Chi-square test

*Mann–Whitney *U* test

Of the minor complications observed (Table 1) in the non-forceps-aided group, soft tissue infection was found in 6 patients (11%), periapical radiolucency due to IMF screw in 1 patient (2%), asymptomatic plate fracture in 1 patient (2%), and poor occlusal need elastic traction in 2 patients (4%). In the forceps-aided group, soft tissue infection was found in 1 patient (1%) and asymptomatic plate fracture in 2 patients (3%).

Of the major complications observed (Table 1) in the non-forceps-aided group, insufficient reduction occurred in 4 patients (7%) and lingual flaring in 1 patient (2%). In the forceps-aided group, non-union occurred in 1 patient (1%) and lingual flaring in 2 patients (3%). The overall complication rate was significantly higher in the non-forceps-aided group (*P* = .004; OR = 4.5, 95% CI, 1.6–12.6).

### Fracture characteristics and reduction techniques

**Fig. 3:**
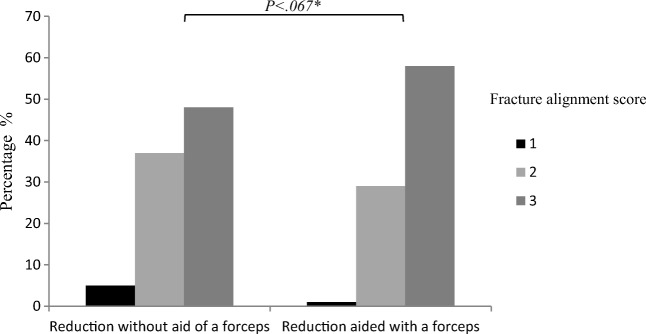
Bar chart comparing the postoperative fracture alignment score by reduction techniques. A score 1—poorly reduced fracture needing reoperation; a score 2—reduced with slight dislocation, but clinically satisfying occlusion; a score 3—reduced with anatomic alignment (*result of Mann–Whitney *U*)

Of the 252 fractures, a total of 179 (71%) primary fractures was treated by ORIF and 73 (29%) concomitant fractures were treated closed (no reduction was needed). The forceps-aided fracture reduction was applied less often in the angle region than in the (para)symphysesal and the body region of the mandible (Table 2). Both simple and comminuted mandibular fractures were as often reduced with and without the aid of forceps. Internal fixation of the fractures was achieved with similar osteosynthesis methods in both groups. Fracture alignment tended to be better for the fractures reduced with the aid of repositioning forceps **(**Fig. 3).

**Table 2:** Fracture characteristics and reduction techniques (unit of analysis: fracture, *n* = 252)

Variables		Open treatment and internal fixation (*n* = 179)		Closed (*n* = 73)	*P* value^c^
		Reduction not aided by the forceps (*n* = 90)^a^	Reduction aided by forceps (*n* = 89)^a^	No reduction (*n* = 73)^a^	
Fracture locations (*n* = 252)	(Para)symphysesal	16 (18)	51 (57)	0	.001
	Body	26 (29)	28 (32)	0	
	Angle	37 (41)	10 (11)	0	
	Ramus	2 (2)	0	21 (29)	
	Condyle	9 (10)	0	52 (71)	
Fracture type (*n* = 252)	Simple	74 (82)	69 (78)	64 (87)	.001
	Comminuted	16 (18)	20 (22)	1 (2)	
	Incomplete	0	0	8 (11)	
Fracture fixation (*n* = 178)^b^	Load sharing	82 (91)	76 (85)		.466
	Load bearing	2 (2)	4 (5)		
	Both	6 (7)	9 (10)		
Complications (*n* = 252)	No	75 (83)	83 (93)	73 (100)	.001
	Yes	15 (17)	6 (7)	0	

^a^Column percentage

^b^Number of valid data (fractures treated with closed treatment excluded due to no reduction was done)

^c^Chi-square test

In the non-forceps-aided group, 15 (17%) fractures had complications (minor *n* = 10, 11%; major *n* = 5, 6%), and in the forceps-aided group, 6 (7%) fractures had complications (minor *n* = 3, 3.5%; major *n* = 3, 3.5%). Detailed complications are shown in Table 3. The overall complications rate was higher in the non-forceps-aided group compared to the forceps-aided group (*P* = .045;OR = 2.7, 95% CI, 1.0–7.4).

**Table 3:** Complications associated with fracture location of each group (*n* = 252 fractures)

Complications		Open treatment and internal fixation (*n* = 179)										Closed (*n* = 73)	
		Reduction not aided by the forceps (*n* = 90)					Reduction aided by forceps (*n* = 89)					No reduction (*n* = 79)	
		(Para)symphyseal	Body	Angle	Ramus	Condyle	(Para)symphyseal	Body	Angle	Ramus	Condyle	Ramus	Condyle
No		13 (82)	20 (77)	31 (83)	2 (100)	9 (100)	50 (98)	27(96)	6 (60)	0	0	0	0
Minor	Infections	0	3 (11)	3 (8)	0	0	0	0	1 (10)				
	Poor occlusion needs elastic traction	1 (6)	1 (4)	0	0	0	0	0	0	0	0	1*	3*
	Asymptomatic plate fracture	0	0	1 (3)	0	0	0	0	2 (20)	0	0	0	0
	Periapical radiolucency due to IMF screw	0	0	1 (3)	0	0	0	0	0	0	0	0	0
Major	Insufficient reduction	1 (6)	2 (8)	1 (3)	0	0	0	0	0	0	0	0	0
	Lingual flaring	1 (6)	0	0	0	0	1 (2)	1 (4)	0	0	0	0	0
	Non-union	0	0	0	0	0	0	0	1 (10)	0	0	0	0

*Expected closed treatment result with a foreseen correction for concomitant (condylar and ramus) fractures

### Confounders

The possible confounding factors for complications are summarized in Table 4. Medical comorbidities were more frequently observed in the fractures reduced by the non-forceps-aided group. However, there was no association between comorbidities and postoperative complications in logistic regression analysis. The fractures of the angle region had a higher complication rate compare to the (para)symphyseal region of the mandible.

**Table 4:** Binary logistic regression analysis: calculation of odds of having postoperative complications

		OR (CI 95%)	*P* value
Unit of analysis number of patients			
Age		1.0 (0.9–1.0)	.927
Gender (male, female)		2.1 (0.7–5.9)	.151
Comorbidity (no, yes)		0.9 (0.1–4.6)	.947
Smoking (no, yes)		0.6 (0.1–2.9)	.544
Oral hygiene (good, poor)		1.2 (0.2–6.5)	.768
Dental status	Complete	Reference	
	Partially	1.5 (0.5–4.1)	.382
	Edentulous	0.5 (0.6–4.9)	.622
Velocity of fracture^a^ (high, low)		1.6 (0.5–4.4)	.364
Condyle fracture (with, without)		0.5 (0.1–1.6)	.305
Fracture	Single	Reference	
	Single condyle	0.4 (0.1–1.5)	.412
	Bilateral	0.6 (0.2–1.9)	.440
	Bilateral condyle	0.7 (0.0–6.8)	.760
Reduction techniques	Forceps aided	Reference	
	Non-forceps aided	4.5 (1.6–12.6)	.004
Unit of analysis number of fractures^b^			
Location	(Para)symphyseal	Reference	
	Body	2.3 (0.6–8.4)	.194
	Angle	4.2 (1.2–14.5)	.021
Fracture type	Simple	Reference	
	Comminuted	0.9 (0.2–3.3)	.900
	Incomplete	0.0	.999
Fixation	Load sharing	Reference	
	Load bearing	0.0	.999
	Both	0.4 (0.6–3.9)	.505
Reduction techniques	Forceps aided	Reference	
	Non-forceps aided	2.7 (1.0–7.4)	.045

^a^Low velocity (fall, assault, sport and home accident); high velocity (motor vehicle and bicycle accident)

^b^Fracture of the condyle and ramus (concomitant) were excluded (treated closed)

## Discussion

Internal fixation of fractures is always preceded by reduction of the fractured fragments. In case of mandibular fractures, this reduction can be achieved by either IMF, manual reduction, and/or using a repositioning forceps [3–5]. In daily practice, reduction starts at the moment IMF is applied or when dislocation is neutralized by hand. Additionally, in more complex or less stable cases, reduction can be facilitated and improved by using reduction forceps.

In this study, we analyzed the fracture alignment and postoperative complications of mandibular fractures reduced with or without the aid of repositioning forceps. The results of this study show that the additional use of a repositioning forceps in the treatment of mandibular fractures, even when they are more complex, result in a better alignment of the fragments and less complications compared to manual repositioning and/or IMF only. These results are in accordance with those of previous studies [2, 10], indicating that using repositioning forceps for the mandibular fracture decreases the postoperative complication rate and provides adequate reduction.

In this study, forceps-aided reduction was mostly applied in the (para)symphyseal and body region and less in the angle. The available forceps are less easy to apply in the posterior region. As this study has shown that a forceps is of additional value in mandibular fracture treatment, there is a need for development of a reduction forceps designed for application in posterior mandibular fractures.

One might expect that repositioning forceps would be used mainly to reduce simple, non-comminuted fractures. In our study, nearly half of the comminuted fractures were also reduced with the aid of repositioning forceps. In these cases, prior to fixation, the larger parts of comminuted fractures were first reduced with repositioning forceps, and then the smaller parts were fitted in. Independently of the reduction technique, complications were more often observed in the angle region compared to (para)symphyseal region of the mandible. This is generally known from literature. Only a small number of fractures in the posterior region were treated with additional use of a forceps (Table 3). Therefore, we are not able to make a clear statement on the additional value of the use of forceps in this specific region.

It has to be mentioned that, in this retrospective study, the method of fracture reduction was selected by the surgeons, which could lead to incorporation of bias. However, the group of mandibular fractures studied was a representative sample of the mandibular fractures treated during the inclusion period. As these fractures are treated by several surgeons in out of office hours, the surgeons could not select the patients or fractures. Therefore, results of this study can be generalized to other surgeons as well. The results show that, irrespective of the use of forceps in simple or comminuted fractures, the application of a reduction forceps favors a positive outcome of mandibular fracture treatment. Mandibular fractures reduced by the aid of repositioning forceps are accompanied by lower complication rates and better alignment.
